# Ventricular tachycardia storm after accidental poisoning of delphinium species: a rare case report

**DOI:** 10.1097/MS9.0000000000001892

**Published:** 2024-03-06

**Authors:** Swikriti Shrestha, Shovit Thapa, Asraf Hussain, Sandesh Lamichhane, Subash Subedi, Sujata KC, Narayan Kandel, Kailash Pant

**Affiliations:** Departments of a Internal Medicine; b Cardiology, Chitwan Medical College Teaching Hospital, Chitwan; c Department of Emergency Medicine, Lubhu Primary Health Center, Lalitpur, Nepal; d Department of Internal Medicine, Division of Cardiovascular Medicine, University of Illinois College of Medicine, OSF Healthcare, Peoria, IL

**Keywords:** case report, delphinium, electrical storm, poisoning, ventricular tachycardia

## Abstract

**Introduction:**

*Delphinium* species are commonly used as medicinal herbs, with a wide range of implications for medical conditions. The injudicious use of this plant has been known to cause devastating side effects, including cardiac arrhythmias.

**Case presentation:**

Here, the authors present an 80-year-old male with incessant ventricular tachycardia after ingestion of this herb. The sinus rhythm was restored after electrical cardioversion and aggressive intravenous antiarrhythmics.

**Clinical discussion:**

To the best knowledge of the authors, no case of a ventricular storm following ingestion of *Delphinium* has been reported till now, probably rendering this case the first one.

**Conclusion:**

This report aims to present the rare case using theoretical concepts from the discipline and to share our approach in the hope of achieving a better understanding of similar cases.

## Introduction

Highlights

- *Delphinium, a* commonly used medicinal herbs in Nepal, is notably utilized for its wide antimicrobial, anti-inflammatory, antineoplastic, antifeedant, and cholinesterase inhibition properties.
- This is a first reported case of *Delphinium* toxicity resulting in fatal arrhythmias such as ventricular storms.
- Clinicians should review a detailed medical history of ingestion of any herbal medicines and rule out the possible cause of herbal intoxication.

‘Nirmasi’, or ‘Nirmashi’, one of the commonly used medicinal herbs in Nepal, is a species that belongs to the genus *Delphinium* of the family Ranunculaceae of which more than 356 species are identified. Nirmasi is infamously used for fever, headache, abdominal pain, jaundice, epilepsy, cough, and skin rashes which may be attributed to its antimicrobial, anti-inflammatory, antineoplastic, antifeedant, and cholinesterase inhibition properties^1,2^. As studies have suggested, the substance responsible for the medicinal properties of *Delphinium* is diterpenoid alkaloids. However, it is a lesser-known fact that the same substances have the potential to cause toxicity with manifestations ranging from gastric irritation to fatal arrhythmias^1,3^. We present a novel case of the occurrence of a ventricular storm after ingestion of *Delphinium* as an herbal treatment for their acidity. The case has been documented as per the SCARE 2020 guidelines^4^.

## Case presentation

An 80-year-old male with no known heart disease presented to the emergency department with decreased mental alertness with a Glasgow Coma Scale of 13/15, respiratory rate of 20 breaths/min, a pulse rate of 170 bpm, blood pressure of 90/60 mmHg, and decreased oxygen saturation. Before the onset of altered mental status, the patient had multiple episodes of vomiting, epigastric discomfort, and tingling in the extremities for 2–3 h. There was no significant past medical history and no history of drug allergy. Family history was insignificant.

**Figure 1:**
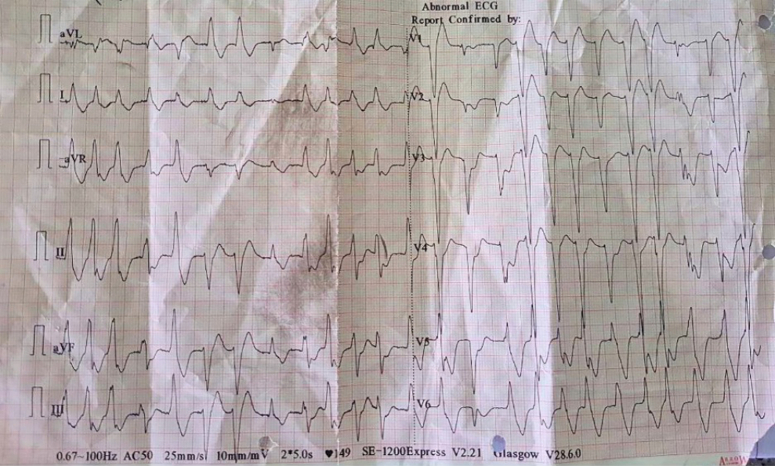
12-lead electrocardiogram showing monomorphic ventricular tachycardia.

His continuous telemetry recorded monomorphic ventricular tachycardia (VT) (Fig. 1) for which bipolar DC shock 150J was delivered along with an immediate cardiology consultation. There was transient reversion of ventricular tachycardia to sinus rhythm with frequent recurrences requiring multiple episodes of repeated DC shocks with increased energy (200J). All the routine blood investigations, urinalysis, and ABG were normal. On detailed interrogation with the patient’s wife found that about 30 min before the onset of the symptoms, the patient had consumed Nirmashi to treat the gastric acidity.

The continuity of care was provided in the coronary care unit where intravenous amiodarone was administered followed by a continuous infusion simultaneously. Intravenous amiodarone followed by continuous infusion was administered simultaneously. In addition, the patient received intravenous calcium gluconate, magnesium sulfate, and sodium bicarbonate. Despite these measures, the patient continued to have episodes of non-sustained VT with varying morphology. So, additional antiarrhythmic drugs were administered (injection lignocaine followed by continuous infusion & intravenous phenytoin). Over 12 h of continuous cardiac monitoring, the arrhythmias were fairly under control, though the patient continued to have low grade II–IVa PVCs for which antiarrhythmics were continued with a titrating dose.

Meanwhile, the results of the routine blood test were consistently normal and troponin level was within the normal limits thus, ruling out the ischaemic changes and electrolyte imbalance (Table 1). The echocardiogram showed an essentially normal study. On the second day of admission, he had episodes of hypotension requiring vasopressor support with Noradrenaline (3 mcg/kg/h), which was down-titrated on subsequent days based on tolerability. Subsequent ECG evaluation showed junctional rhythms, after which the antiarrhythmic drugs were discontinued. Gradually, sinus rhythm was restored with first-degree AV block, which recovered on subsequent days. However, the toxicological reports of the ingested herbal medicine could not be documented due to the lack of specific detection methods.

**Table 1:** Baseline investigation on the day of presentation

Lab parameters	Values on presentation (reference range)
Haemoglobin (gm/dl)	11.4 (12–16)
WBC count (/mm^3^)	7500 (4000–11 000)
Platelets (/mm^3^)	217 000 (150 000–400 000)
Random blood glucose (mg/dl)	123 (70–140)
Sodium (mmol/l)	141 (135–150)
Potassium (mmol/l)	4.5 (3.5–5.5)
Calcium (mg/dl)	9.28 (8–11)
Magnesium (mg/dl)	2.4 (1.8–2.4)
Blood urea (mg/dl)	28.59 (15–45)
Creatinine (mg/dl)	1.09 (0.4–1.4)
Total bilirubin (mg/dl)	0.58 (0.2–1)
ALT (IU/l)	22.57 (<45)
AST (IU/l)	33.34 (<40)
Alkaline phosphatase (U/l)	83.5 (50–136)
Troponin [quantitative] (ng/ml)	0.02 (<0.1)

ALT, alanine transaminase; AST, aspartate transaminase; WBC, white blood cell.

**Figure 2:**
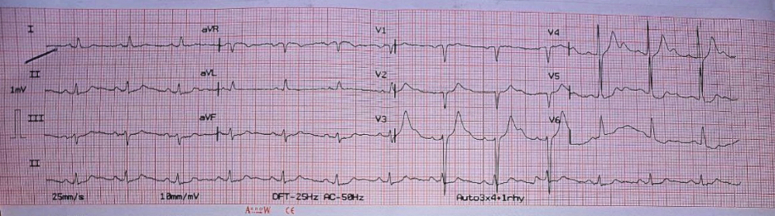
12-lead electrocardiogram showing normal sinus rhythm on the third day of admission.

The patient was then discharged on day 6 of admission. The patient followed up after a week and a month of discharge and was asymptomatic. His repeat ECG showed sinus rhythm with no evidence of arrhythmias (Fig. 2).

## Discussion

Herbal medicine has been studied for its role in the treatment of cardiovascular disease since time immemorial and has become the mainstay of human pharmacotherapy. However, many herbal remedies have the potential to cause serious toxic effects and major drug-to-drug interactions^5^. The spectrum of heart toxicities included hypertension, hypotension, hypokalemia, bradycardia, tachycardia, arrhythmia, ventricular fibrillation (VF), heart attack, cardiac arrest, heart failure, and death^6^. Most of the wild flora of Nepal is rich in medicinal properties out of which *Delphinium brunonianum* has been popularly used for the treatment of many ailments like fever, headache, abdominal pain, jaundice, cough, toxin removal, and skin rashes. However, some species of *Delphinium* are toxic depending on the chemical constituents of the plant^2^. Tomassoni *et al.* ^7^ reported the cardiotoxic nature upon consumption of *Delphinium* manifested as VT. Another study conducted in the Manang district of Nepal reported a case of hypotension and sinus bradycardia after ingestion of *Delphinium* species^2^. The index case had accidental poisoning upon consumption of *Delphinium* manifesting from gastric upset to a severe form of cardiotoxicity that is ventricular storm.

Ventricular storm is itself a life-threatening syndrome, a state of cardiac electrical instability with three or more episodes of VT or VF in a short period of time, typically within 24 h^8^. Most ventricular storms have underlying structural heart disease associated with drug toxicity, electrolyte imbalances, heart failure, ischaemia, and/or reperfusion, among other factors. A common pathway in patients with risk factors is the enhanced sympathetic catecholaminergic overdrive, which can augment and perpetuate the arrhythmia itself. The first step in management is to identify the initial rhythm and hemodynamic status of the patient. Unstable patients may require cardioversion and defibrillation. Thus, the treatment of the ventricular storm is to look for the potential causes of the ventricular storm along with the implementation of aggressive abortive interventions^9^.

In our index case, laboratory workup showed no electrolyte imbalances, transthoracic echocardiography was normal with LVEF 60%. The temporal relationship between the patient’s history of consumption of Delphinium and the development of the ventricular storm gives us an idea of the possible cardiotoxic nature of the Delphinium species. No specific antidote for *Delphinium* poisoning in humans has been identified to this date^2^, for which the patient had undergone aggressive abortive measures.

## Conclusion

This study on accidental poisoning emphasizes further study on the safety and efficacy of *Delphinium* species and awareness among the general population and clinicians about the implications of herbal medicine. Our case report presents the onset of arrhythmia upon consumption of Nirmasi on the exclusion of various potential risk factors prompting the high evidence research to study the correlation between *Delphinium* species and cardiovascular condition.

## Ethical approval

This case report did not require review by the institutional review committee.

## Consent

Written informed consent was obtained from the patient for the publication of this case report and accompanying images. A copy of the written consent is available for review by the Editor-in Chief of this journal on request.

## Source of funding

The authors declare that this work was not supported by any grants.

## Author contribution

S. Shrestha: concept of the study, collection of data, literature review and writing the manuscript. S.T.: literature review revising and editing the manuscript. A.H.: literature review, revising and editing the manuscript. S.L.: literature review, and writing the manuscript. S. Subedi: literature review and writing the manuscript. S.K.C.: literature review and writing the manuscript. N.K.: literature review, revising and editing the manuscript. K.P.: literature review, revising and editing the manuscript. All authors were involved in drafting and revising the manuscript, and approved the final version.

## Conflicts of interest disclosure

All the authors declare that they have no competing interests.

## Research registration unique identifying number (UIN)

Not applicable.

## Guarantor

Mr. Sandesh Lamichhane.

## Data availability statement

All the data generated during the current study are publicly available.

## Provenance and peer review

Not commissioned, externally peer-reviewed.
